# Neoantigen Specific T Cells Derived From T Cell-Derived Induced Pluripotent Stem Cells for the Treatment of Hepatocellular Carcinoma: Potential and Challenges

**DOI:** 10.3389/fimmu.2021.690565

**Published:** 2021-05-13

**Authors:** Fei Lu, Xiao-Jing-Nan Ma, Wei-Lin Jin, Yang Luo, Xun Li

**Affiliations:** ^1^ The First School of Clinical Medicine, Lanzhou University, Lanzhou, China; ^2^ Department of General Surgery, The First Hospital of Lanzhou University, Lanzhou, China; ^3^ Key Laboratory of Biotherapy and Regenerative Medicine of Gansu Province, The First Hospital of Lanzhou University, Lanzhou, China; ^4^ Institute of Cancer Neuroscience, Medical Frontier Innovation Research Center, The First Hospital of Lanzhou University, Lanzhou, China; ^5^ Hepatopancreatobiliary Surgery Institute of Gansu Province, The First Hospital of Lanzhou University, Lanzhou, China; ^6^ Health Science Center, Lanzhou University, Lanzhou, China; ^7^ Medical Frontier Innovation Research Center, The First Hospital of Lanzhou University, Lanzhou, China

**Keywords:** hepatocellular carcinoma, immunotherapy, neoantigen, T cell-derived induced pluripotent stem cells, T cell receptors, T cells

## Abstract

Immunotherapy has become an indispensable part of the comprehensive treatment of hepatocellular carcinoma (HCC). Immunotherapy has proven effective in patients with early HCC, advanced HCC, or HCC recurrence after liver transplantation. Clinically, the most commonly used immunotherapy is immune checkpoint inhibition using monoclonal antibodies, such as CTLA-4 and PD-1. However, it cannot fundamentally solve the problems of a weakened immune system and inactivation of immune cells involved in killing tumor cells. T cells can express tumor antigen-recognizing T cell receptors (TCRs) or chimeric antigen receptors (CARs) on the cell surface through gene editing to improve the specificity and responsiveness of immune cells. According to previous studies, TCR-T cell therapy is significantly better than CAR-T cell therapy in the treatment of solid tumors and is one of the most promising immune cell therapies for solid tumors so far. However, its application in the treatment of HCC is still being researched. Technological advancements in induction and redifferentiation of induced pluripotent stem cells (iPSCs) allow us to use T cells to induce T cell-derived iPSCs (T-iPSCs) and then differentiate them into TCR-T cells. This has allowed a convenient strategy to study HCC models and explore optimal treatment strategies. This review gives an overview of the major advances in the development of protocols to generate neoantigen-specific TCR-T cells from T-iPSCs. We will also discuss their potential and challenges in the treatment of HCC.

## Introduction

**Figure 1:**
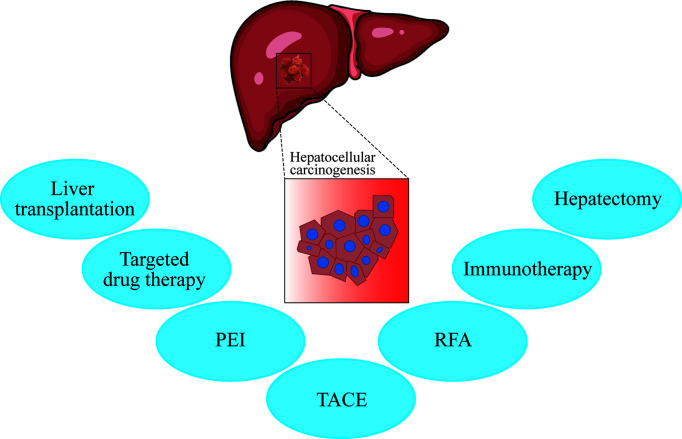
The current treatments for liver cancer include hepatectomy, immunotherapy, transcatheter arterial chemoembolization (TACE), radiofrequency ablation (RFA), percutaneous ethanol injection (PEI), targeted drug therapy (sorafenib/FOLFOX4), and liver transplantation (UCSF standard). According to the patient’s condition, such as the degree of liver cirrhosis, liver function (child grade), number of tumors, size of tumors, and metastasis, clinicians select the appropriate treatment plan or perform combination therapy.

Hepatocellular carcinoma (HCC) is the sixth most common type of cancer worldwide and the third leading cause of cancer-related death, with approximately 50% of HCC cases occurring in China (1). Localized stage HCC has a five-year relative survival rate in near 31% of patients with HCC, in all races (2). About 70% of HCC cases are diagnosed at the advanced stage (3, 4), which requires systemic treatment. Surgical treatment is considered the most comprehensive treatment for HCC. Non-surgical treatments mostly include local treatments such as transcatheter hepatic arterial chemoembolization (TACE), radiofrequency ablation (RFA), and percutaneous ethanol injection (PEI). For mid-late stage HCC, molecular targeting drugs such as Sorafenib are currently the most commonly used treatment (**Figure 1**).

While earlier studies were focused on the tumor parenchyma, currently, the research focus has shifted to the tumor stroma and tumor microenvironment. The microenvironment of solid tumor usually contains a large number of stroma and immunosuppressive cells, making it difficult for the immune cells to attack the tumor; the hypoxic environment and nutritional deficiency also hinder the proliferation and survival of T cells (5). Thus, the immune system is unable to clear the tumor. Adoptive cell therapy (ACT) might be able to overcome these challenges. It improves the patient’s immune environment, reactivating and proliferating the non-functional immune cells, allowing them to kill the tumor cells in the patient’s body.

Tumor immunotherapy has attracted much attention because of its ability to enhance the body’s autoimmunity to kill tumors. In addition to adoptive cell therapy, immune-based therapies for HCC have been used in clinical applications or are undergoing clinical trials. Immune-based therapies include methods such as immune checkpoint inhibitors, inhibitory cytokine blockers, oncolytic viruses, and genetic vaccines (6). Studies have shown that after administration of tremelimumab, an antibody targeting CTLA-4, combined with radiofrequency ablation to treat advanced liver cancer, led to a significant increase in CD8+ T cells in the cancer tissue of patients, significantly prolonging the progression-free survival period (7). Monoclonal antibodies that target immune checkpoints have achieved remarkable therapeutic results in different types of tumors, but it also has several challenges, for example, controlling immune-related adverse reactions and applying combination therapy strategies. Moreover, this type of immunotherapy is time-consuming and slow in onset. Further research is needed to design and optimize such immunotherapeutic treatment strategies.

With respect to ACT, chimeric antigen receptor (CAR)-T cell therapy and T cell receptor (TCR)-T cell therapy are most commonly used. T cells express TCRs or CARs that recognize tumor antigens on the cell surface through gene editing technology, rendering T cells the specificity in their functions. Moreover, compared with cumbersome screening to obtain tumor infiltrating lymphocytes (TILs), the acquisition and preparation of genetically engineered T cells is simpler (8). With the leukemia treatment trial of CAR-T cell therapy conducted by the University of Pennsylvania, it has begun to be widely used in the treatment of tumors (9). The development of CAR-T cell therapy has achieved clinical cures for certain types of tumors in the blood, like B-cell leukemia and lymphoma. Although CAR-T cells have achieved remarkable results in the treatment of certain types of leukemia, their use in treating solid tumors faces several challenges. Therefore, the successful application of TCR-T cell therapy in treating solid tumors makes it one of the most promising immune cell therapy methods for solid tumors, e.g., the use of NY-ESO-1 antigen-specific TCR-T cells against some tumors like melanoma, synovial sarcoma, multiple myeloma and disseminated neuroblastoma (10–13).

TCR-T cell therapy is widely used in tumor therapy because of its wide range of therapeutic targets, high affinity, and high specificity for tumor antigen. However, it also elicits adverse effects, especially off-target effects and severe untargeted toxicity (14–17). Induced pluripotent stem cells (iPSCs) emerged as a viable tool to derive T cells owing to their plasticity, high proliferative capacity, and no ethical restrictions. Therefore, developing a model of neoantigen T cell-derived iPSCs (T-iPSCs) is increasingly being considered in cancer precision medicine, tumor immunotherapy and regenerative medicine, to study their safety and efficacy in treating HCC. Several studies are underway to develop protocols to generate personalized and specific TCR-T cells using T-iPSCs. This review will describe in detail the recent advances made in developing TCR-T cells using T-iPSCs. We will also discuss the potential applications and challenges of using neoantigen T cells derived from T-iPSCs for the treatment of HCC.

## Importance of Neoantigen Specific T Cells Derived From T-iPSCs in the Treatment of HCC

Traditional hepatectomy is effective in treating early HCC but not for advanced HCC and metastatic cancer cells. Radiotherapy and chemotherapy have poor selectivity for liver cancer, resulting in damage of normal tissues while killing cancer cells. Although targeted drugs such as sorafenib, donafenib, atezolizumab, and bevacizumab are effective in treating HCC, they cannot suppress the mutation and drug resistance that develops in tumor cells. Extensive research has been carried out to develop drugs to treat tumors. However, molecular-targeted drugs have their own limitations due to drug resistance, side effects, lack of tumor specificity, and unsustained tumor killing activity. Therefore, some patients do not benefit from systemic treatment with molecular-targeted drugs. This challenge can perhaps be overcome by tumor immunotherapy and regenerative medicine.

Immunotherapy does not require surgery; immune cells can be targeted to the tumor through direct injection or through the peripheral. Most tumor patients are in a state of immunosuppression. Therefore, immunotherapy is necessary, to change the overall immune state of the patient or to change the local immune microenvironment of the tumor.

In China, HCC cells usually have integrated HBV-DNA fragments (18) and can therefore synthesize a peptide chain which can bind to major histocompatibility complex (MHC) molecules, enabling it to be recognized by T cells. Hepatitis B surface antigen (HbsAg) may be used as a potential target for TCR-T cell therapy (19). TCR genes can specifically recognize and kill HepG2 tumor cells *in vitro* through CD8+ T cells. They can also eliminate HepG2 tumors in NSG mice (15). Hafezi et al. showed that HBV-specific TCR-T cells have potential applications in organ transplantation patients with recurring HBV-HCC (20). These results indicate that TCR-T cell therapy is efficient in the treatment of solid tumors such as HCC, and adoptive cell transfer therapy of liver cancer might eventually become the optimal solution in its comprehensive treatment.

**Figure 2:**
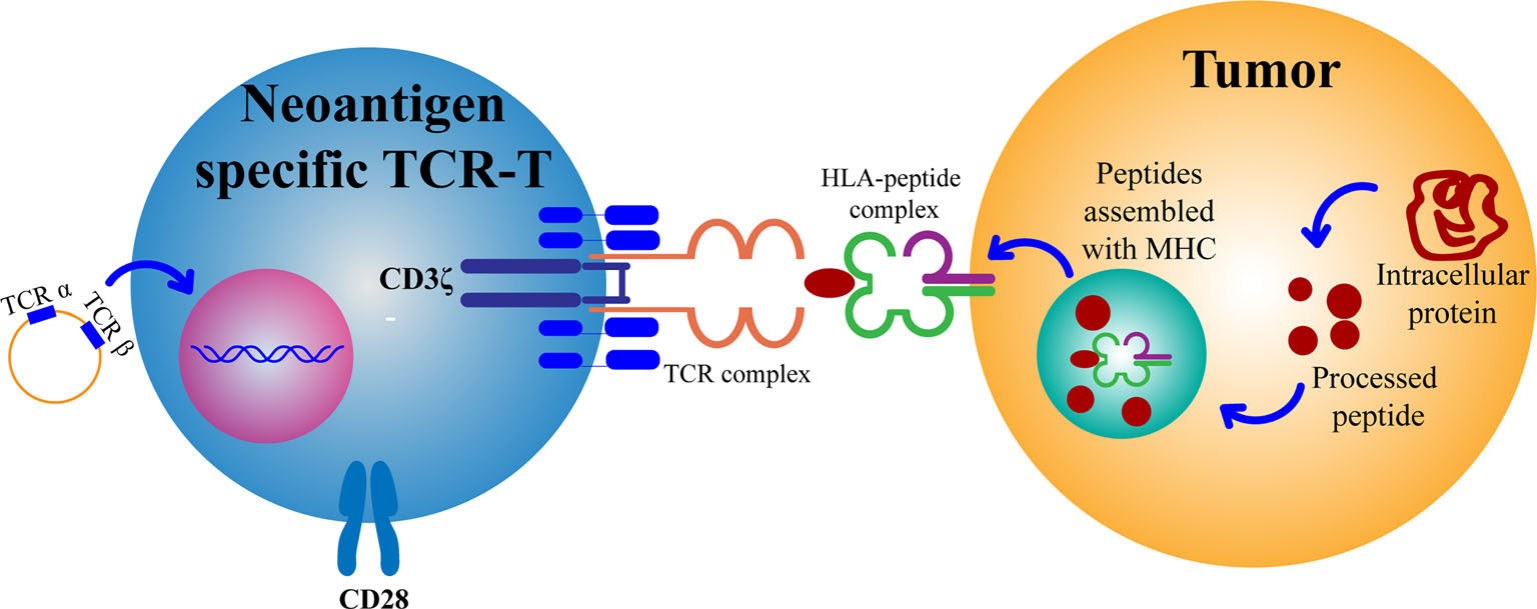
Mature CD8+ T cells with a new antigen-specific TCR gene can recognize antigen targets and thus attack and kill tumor cells. TCR, T cell receptor; HLA, human leukocyte antigen; MHC, major histocompatibility complex.

The selection of TCR targets is a key challenge in TCR-T cell therapy. Generally, there are two criteria for determining target tumor antigens. First, if the antigen is only expressed in tumors; second, if there are enough patients who can benefit from this treatment. Thus, there are three tumor antigens that can be used as suitable targets for TCR-transduced T cell therapy, including cancer-testis antigens, oncogenic virus antigens, and neoantigens. Tumors produce mutated peptides due to accumulated somatic mutations. MHC molecules can bind to such antigen peptides to form new antigens on the cell surface (**Figure 2**). These neoantigens are usually unique to cancer cells and individuals. Therefore, they are ideal targets for designing T cell immunotherapy. Neoantigens may also become potential targets for developing immunotherapy in patients with other cancer types (19).

## Development of TCR-T Cell Targeting Neoantigens Using T-iPSCs

The production of TCR-T cell targeting neoantigens requires three steps: screening neoantigens, constructing T-iPSCs, and inducing TCR-T cells.

### Screening Neoantigens

According to existing literature, tumor rejection epitopes may be derived from two classes of antigens. The first class is developed from non-mutated proteins to which T cell tolerance is incomplete because of their restricted tissue expression pattern. The second class can be developed from peptides that are entirely absent from the normal human genome, the so called neoantigens (21). Neoantigens are derived from antigens produced by the mutant protein, and antigens produced by the integration of the tumorigenic virus into the human genome. Neoantigens do not cause central immune tolerance and have strong immunogenicity. There are two conditions for screening neoantigens: first, a mutated protein needs to be processed and then presented as a mutant peptide by MHC molecules; second, T cells that can recognize this peptide-MHC complex should be present (21).

All malignant tumors harbor non-synonymous mutations or other genetic alterations (22). This makes it feasible to identify mutant antigens by technical means. Immunogenic antigenic peptides were identified using next-generation sequencing technology in conjugation with bioinformatics, which predicted epitope peptides binding mutated proteins to human leukocyte antigen (HLA) with high affinity, and stimulated lymphocytes to detect cytokines *in vitro*. Robbins et al. developed a new screening approach involving mining whole-exome sequence data to identify mutated proteins expressed in the tumor of the patient. They then synthesized and evaluated candidate mutated T cell epitopes that were identified using an MHC-binding algorithm for being recognized by TILs. This simplified approach for identifying mutated antigens recognized by T cells avoids the need to laboriously develop and screen cDNA libraries of tumors and may be easier for practical application (23).

Based on whole-exome sequence, using non-synonymous mutations, a tandem minigene (TMG) vector was designed and synthesized, which was transcribed into multi-epitope RNA *in vitro* and transferred to antigen-presenting cells. Subsequently, T cells were stimulated by these antigen-presenting cells. This enables identification of immunogenicity epitopes of RNA and specific mutant epitopes of immunogenicity, as needed. Tran et al. used whole-exome sequence which revealed 26 non-synonymous mutations in a widely metastatic cholangiocarcinoma case. The study used minigene approach wherein multiple minigenes were synthesized in tandem to generate TMG constructs. These constructs were then used as templates for the generation of *in vitro* transcribed (IVT) RNA. Each of these IVT TMG RNAs was then individually transfected into autologous antigen-presenting cells, followed by coculture with TILs. The reactivity predominated in the CD4+ T cell population, as demonstrated by up-regulation of the activation markers OX40 and 4-BB. The study successfully identified HLA-DQB1*0601 restrictive REBB2IP neoantigen sequence NSKEETGHLENGN (24).

Using bioinformatics, multiple antigenic peptide fragments were designed for hotspot mutations of high-frequency mutated genes, and the optimal epitopes were screened *in vitro*, so as to cover more patients with mutations of this gene site. Schumacher et al. demonstrated that isocitrate dehydrogenase 1 (IDH1) (R132H) contains an immunogenic epitope suitable for mutation-specific vaccination. Peptides encompassing the mutated region are presented on MHC class II and they induce mutation-specific CD4+ helper T cell-1 (TH1) responses in gliomas (25). These methods can efficiently screen neoantigens, thus laying the foundation for the subsequent production of neoantigen specific TCR-T cells.

### T-iPSCs

Dedifferentiation of adult cells into stem cells was first proposed by Japanese scholar professor Shinya Yamanaka in 2006. Their team proposed that differentiated cells from mouse embryo or adult fibroblasts can be reprogrammed into iPSCs by introducing four transcription factors, Oct3/4, Sox2, c-Myc, and Klf4 (26). In 2007, Takahashi et al. found that iPSCs developed from adult human dermal fibroblasts using Oct3/4, Sox2, c-Myc, and Klf4 (OSKM) were similar to human embryonic stem cells (ESCs) and could differentiate into cell types of the three germ layers (27). In 2008, Park et al. developed a protocol using the four transcription factors (Oct3/4, Sox2, c-Myc, and Klf4) to develop iPSCs from reprogramming of human somatic cells. They found that only Oct3/4 and Sox2 were necessary in such a case, and NANOG and LIN28 can replace c-Myc and Klf4, respectively. Adding human telomerase reverse transcriptase (hTERT) and SV40 Large T antigen can improve the efficiency of reprogramming the cells during the cultivation process. They also pointed out that human iPSCs resemble ESCs not only in morphology and gene expression but also in the capacity to form teratomas in immune-deficient mice. The study also established a method to yield patient-specific cells that may be cultured *in vitro* (28). In 2009, Hamilton et al. used a lentiviral transduction system induced by doxycycline and four transcription factors (OSKM) to reprogram mouse embryonic fibroblasts into iPSCs (29). Since then, iPSCs have been widely studied by several researchers, which will pave way for research into treatment methods for various diseases (30).

**Figure 3:**
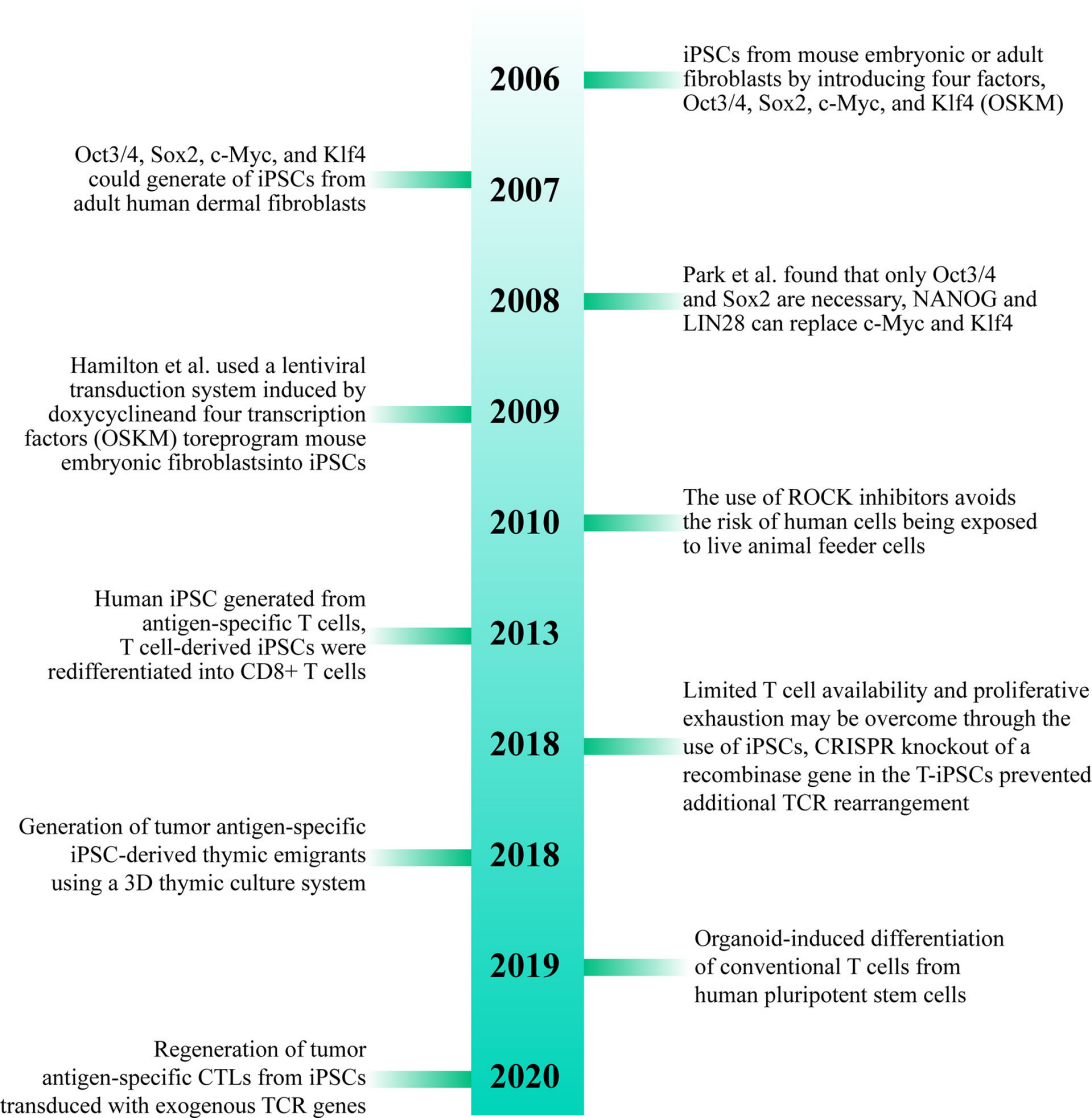
Brief history of T cell-derived induced pluripotent stem cells (T-iPSCs). TCR, T Cell Receptor; iPSCs, induced pluripotent stem cells; CTLs, cytotoxic T lymphocytes.

More than ten years after the advent of iPSCs, a variety of methods for inducing iPSCs *in vitro* have been studied and improvements have been made to the original method. For example, the use of ROCK inhibitors can improve the efficiency and survival rate of human iPSCs (31). The use of episomes and HDAC inhibitors can lead to efficient production of iPSCs from peripheral blood (32). It was found that reprogramming of adult fibroblasts and mouse embryos into iPSCs using serum-containing media is often not as effective as using serum-free media (33). Some studies have shown that iPSCs can also be successfully developed without c-Myc, but it results in low efficiency and delay in development of iPSCs (34, 35) (**Figure 3**).

The iPSCs induced by T cells are called T-iPSCs, which retain the assembled “endogenous” TCR gene after nuclear reprogramming. Notably, T cells differentiated from T-iPSCs have the same arrangement pattern of TCR genes as original T cells. This makes T-iPSCs an efficient raw material to be used for the propagation of numerous T cells, all of which express antigen-specific TCRs (36, 37).

However, Minagawa et al. discovered that T cells had lost their antigen specificity due to the following factors: (i) there is a rearrangement of the TCR gene at the double positive (DP) stage in the CD8*αβ*+ T cells generated from human T-iPSCs. The researchers used CRISPR technology to knock out the recombinase gene (RAG2) in T-iPSC to prevent this. (ii) During the treatment stage, they also found that the exogenous and the endogenous TCR chains were paired in some T cells (38). To solve this problem, a single viral vector encoding two TCR chain genes can be used in the viral transfection stage, which will reduce the probability of mismatch between the exogenous TCR chain and the endogenous TCR chain (39). Other studies have used siRNA to down-regulate endogenous CD3, or zinc finger nuclease knockout technology to knock out endogenous TCR to reduce the probability of mismatches (40, 41).

T cells used to induce T-iPSCs are mainly derived by isolating autologous TILs from resected tumors of patients and obtaining peripheral lymphocytes (42). The application of T-iPSC to generate a large number of tumor antigen-specific T cells solves the problem of short lifespan of activated cytotoxic T lymphocytes (CTLs) in traditional tumor immunotherapy. Normal T cells induced by the antigen presenting cells *in vitro* will be inactive in a short period, and therefore, cannot effectively attack tumors (43).

### Neoantigen Specific T-iPSCs

The unit of TCR-T executive function is the TCR-CD3 complex, which belongs to the immune protein superfamily, a heterodimer consisting of *α*- and *β*-chains. The peptide chain is divided into three parts, the extracellular region, the cellular transmembrane region, and the intracellular region. Moreover, neoantigen specific TCR *α*- and *β*-chains are the major components of TCR-T cells. A study identified neoantigen specific TCR *α*- and *β*-chains and inserted them into T-iPSCs. After successfully inducing the rearranged TCR gene into iPSC, in the preselection stage, it was able to effectively produce cells which expressed TCR on the surface (43). Compared with the ordinary T-iPSCs, the TCR-iPSCs with the new antigen inserted in them solved the problem of the heterogeneity of the generated T cells. The TCR-iPSC clone produced is simple, fast, and high-quality (44).

A study showed that using gene expression vectors, TCR *α*- and *β*-chains transduced into human T lymphocytes could mediate tumor regression. This has been applied by researchers for clinical trials in humans, and no adverse events have been observed so far (11, 17).

According to a previous study, the steps to generate neoantigen specific TCR α chains and β chains are as follows: Tumor reactive T cells derived from TILs and CTLs of peripheral blood from patients and T cells by autologous antigen presenting cells (APCs) are loaded with synthetic tandem minigene (TMG) or peptides encoding mutated antigens. After that, the newly acquired T cells are identified. Reactive T cells are identified based on the expression of active molecules such as 4-1BB on CD8+ T cells and OX-40 on CD4+ T cells, and then purified and amplified using flow cytometry (24). A new method was designed by Cohen at al. for the identification, isolation, and utilization of neoantigen-specific T cells. They used neoepitope MHC/peptide tetramers to combine T cells in the fresh tumor digests and peripheral blood to obtain neoantigen-specific T cells (45, 46). A small number of neoantigen-specific T cells were successfully obtained using peptide-MHC polymerization method, enabling peripheral blood mononuclear cell (PBMC) to become a potential source of neoantigen epitope-specific TCR. Then sequencing of TCR, obtain Neoantigen specific TCR *α* chains and *β* chains.

### Neoantigen Specific TCR-T Cells

The most critical step is to induce differentiation of neoantigen-specific T-iPSC into mature TCR-T cells. These specific TCR-T cells are monoclonal and highly accurate in cell targeting. Unlike the exogenous TCR transfer technology to induce T cells from hematopoietic stem cells and peripheral blood into tumor antigen-specific T cells, the neoantigen-specific TCR-T cells do not generate mismatched TCR, reducing unnecessary issues in screening and treatment (36). Generally, the induction of lymphoid cells is achieved by constructing a thymic microenvironment in which T cells proliferate. To generate T cells from iPSCs, it needs to be induced into mesoderm, and needs to perform hematopoietic functions (47). Common methods are as follows:

Differentiated and developed CD3+ T cells (3–5) × 10^5^ were extracted from PBMC using magnetic beads, and T cells were stimulated in T cell medium with leben-CD3/CD28 magnetic beads and IL-2. The T cell culture medium contained 20 ng/ml IL-2, 10 ng/ml IL-7, and 10 ng/ml IL-15. On the second day after stimulation, oct3/4, SOX2, KLF4, and c-MYC transcription factors packaged with sendai virus were transfected into T cells. Rat embryonic fibroblasts were laid on the 8^th^ day, and the medium containing 5 ng/ml basic fibroblast growth factor (b-FGF) human induced pluripotent stem cells was replaced 12 days after culture. T-iPSCs were collected at 33–40 days. Subsequently, in the presence of bone marrow mesenchymal stem cells (C3H10T1/2), vascular endothelial growth factor (VEGF), stem cell factor (SCF), and Tyrosine Kinase ligand 3 (FLT-3L). iPSCs differentiated into mesodermal CD34+ hematopoietic stem cells and hematopoietic progenitor cells. Cells were transfected with OP9-DL1 FLT-3 and IL-7 culture medium at day 14 to obtain T cell lineage (36).

**Figure 4:**
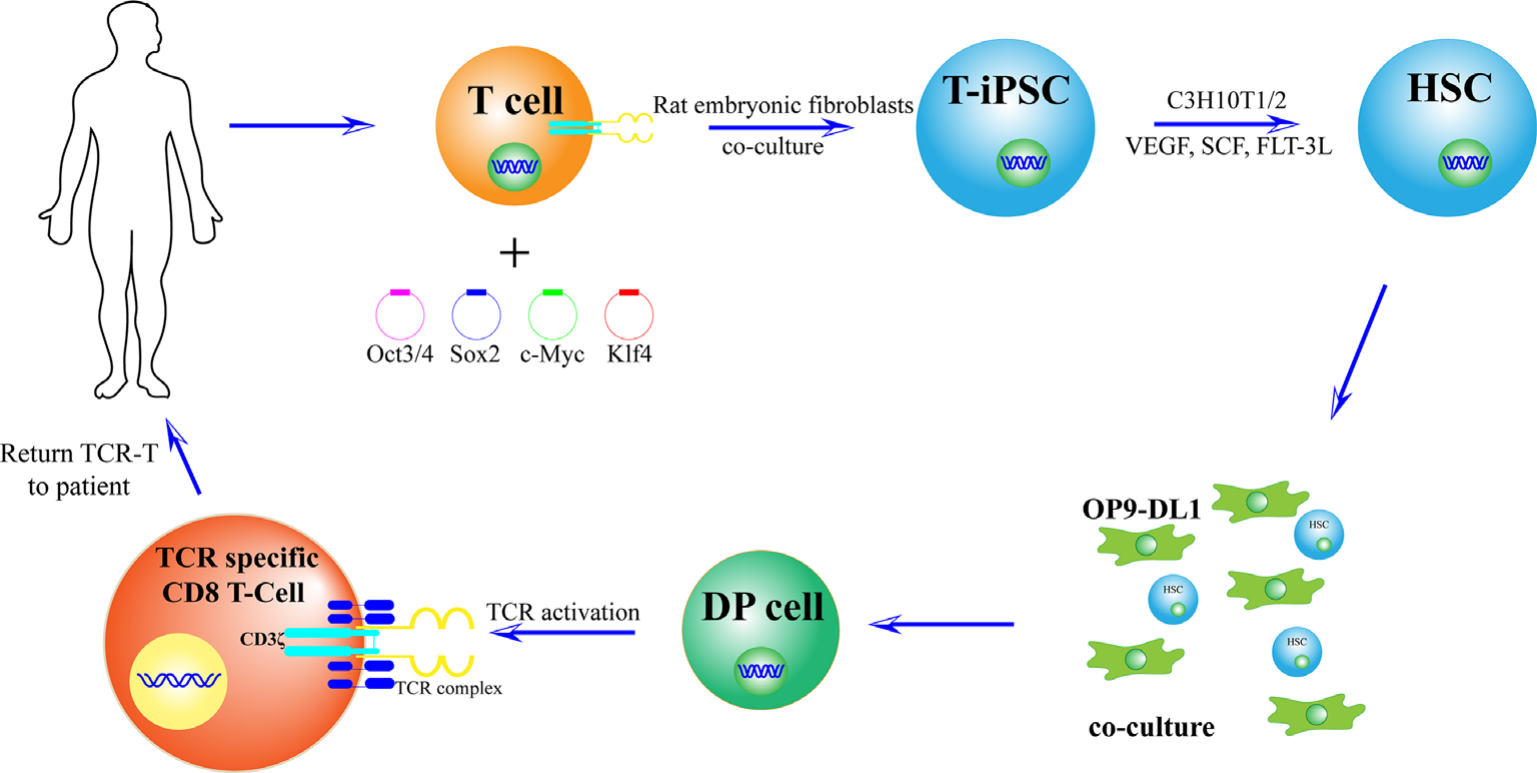
Preparation of new antigen-specific TCR-T cells. First, T cells with new antigen-specific TCR rearrangement gene were isolated from the body. Second, after adding OCT3/4, Sox2, KLF4, and c-MYC, T cells were induced to T-iPSC. Third, in the presence of bone marrow mesenchymal stem cells (C3H10T1/2), vascular endothelial growth factor (VEGF), stem cell factor (SCF), and tyrosine kinase ligand 3 (FLT-3L), T-iPSCs were differentiated into mesodermal CD34+ hematopoietic stem cells (HSCs) and hematopoietic progenitor cells. Cells were transfected to OP9-DL1 FLT-3 and IL-7 culture medium to obtain T cell lineage. Thus, a large number of T cells will be used to recognize and kill tumor cells.

Vizcard et al. showed that a 3D thymic culture system enables the generation of a homogeneous antigen-specific T cell subset. These T-cells generated a unique product which was named iPSC-derived thymic emigrants (iTEs); they closely mimic naïve T cells and exhibit potent anti-tumor activity. The study has designed the 3D thymic culture method based on the traditional fetal thymic organ culture (FTOC) system (48). Another study proved that by co-cultivation of OP9-DL1/DL4 cells which express the Notch ligand, TCR-iPSC inserted with neoantigens can induce production of tumor-specific CD8+ T cells (49) (**Figure 4**).

## Potential and Challenges of TCR-T Cell Induced by T-iPSCs for the Treatment of HCC

### Antigen Selection

Among the current TCR-T cell therapies, TCR-T cells with cancer testis antigen (CT) as the target are the most commonly used. CT is expressed in a variety of tumor tissues, but not in normal tissues other than testis, placenta, and fetal ovaries. Because of its unique expression, it has become an ideal target antigen for tumor immunotherapy. The most used antigen target is NY-ESO-1.

NY-ESO-1 is a tumor-sharing antigen screened from esophageal cancer cDNA expression library using serological analysis of recombinant cDNA library (50). The frequency of expression of the antigen NY-ESO-1 in various tumors is different, and so is the frequency of protein expression. The descending order of protein expression in various tumors is: neuroblastoma, synovial sarcoma, and malignant melanoma. The NY-ESO-1 mRNA is highly expressed in prostate cancer, bladder cancer, breast cancer, multiple myeloma, and hepatocellular carcinoma, and has low expression in oral squamous cell carcinoma and esophageal cancer (51–55). In 2011, it was first reported that TCR-T cell therapy targeting NY-ESO-1 was effective in both melanoma and synovial sarcoma (10). The use of lentivirus-mediated TCR-T cells is also effective in the treatment of hematological malignancies like multiple myeloma (17).

While TCR-T cell therapy has achieved success in other cancers, it is at the clinical trial stage in liver cancer treatment. Currently, there are 6 such clinical trials underway for the treatment of HCC (https://www.clinicaltrials.gov) [Accessed March 15, 2021] (refer to **Table 1** for details).

**Table 1:** Recent clinical trials related to TCR-T cells for the treatment of HCC.

Clinical Trial	NCT number	Host/Country
Redirected HBV-specific T Cells in patients with HBV-related HCC (SAFE-T-HBV) (SAFE-T-HBV)	NCT04745403	Singapore General Hospital, Singapore
TCR-redirected T cell infusion in subjects with recurrent HBV-related HCC post liver transplantation	NCT02719782	The Third Affiliated Hospital of Sun Yat-sen University, Guangzhou, Guangdong, China
TCR-redirected T cell treatment in patients with recurrent HBV-related HCC post liver transplantation	NCT04677088	The First Affiliated Hospital of Sun Yat-sen University, Guangzhou, Guangdong, China
TCR-redirected T cell infusions to prevent HCC recurrence post Liver transplantation	NCT02686372	The First Affiliated Hospital of Sun Yat-sen University, Guangzhou, Guangdong, China
Autologous CAR-T/TCR-T cell immunotherapy for solid malignancies	NCT03941626	Henan Provincial People’s Hospital, Zhengzhou, Henan, China
Autologous CAR-T/TCR-T cell immunotherapy for malignancies	NCT03638206	The First Affiliated Hospital of Zhengzhou University, Zhengzhou, Henan, China

HBV, Hepatitis B Virus; HCC, Hepatocellular Carcinoma; TCR, T Cell Receptor; CAR-T, Chimeric Antigen Receptor T-Cell; TCR-T, T cell receptor T-Cells.

Although adoptive immunotherapy is relatively new, tumor-specific antigens have been studied for more than 50 years. However, the prolonged research and high investment in this field have brought about very few results. In adoptive immunotherapy there is always a need for suitable immune attack targets (16).

In the past, TCR-T cell therapies targeted tumor-associated antigens (TAAs), which are also expressed in normal cells. Therefore, after treatment with genetically engineered T cells targeting carcinoembryonic antigen (CEA), patients have experienced severe transient colitis (56). Patients receiving MART-1 and gp100 TCR treatment also showed toxicity in the skin, eyes, and ears (57). TCR-T cells targeting MAGE-A12 also produced severe neurotoxicity during the treatment process and caused mortality in patients (58).

Although TAAs have many side effects in targeted therapy, they have other advantages. In recent years, many studies have confirmed that the immune system can detect TAAs, and thus, the serum of patients with cancer contains the respective antibodies (59). Therefore, the anti-TAA/TAA system is of great significance as an early cancer biomarker and for predicting the prognosis of the disease. For example, when anti-TAAs are added as a diagnostic marker combined with alpha-fetoprotein (AFP), the sensitivity and specificity to diagnose HCC are improved, compared with AFP alone (60). Wu et al. used alpha-fetoprotein combined with tumor size and alkaline phosphatase levels to invent a simpler and more efficient prognostic scoring system for predicting overall survival (OS) and disease-free survival (DFS) of patients with spontaneously ruptured liver cancer (60). It was proved that the use of multiple antigen microarrays to detect autoantibodies of TAAs can detect and diagnose cancers (61). Wang et al. proposed that anti-TAA autoantibodies may appear in the early stage of HCC, which can be used as a serological marker for early screening of HCC, and it also provides a new way for HCC detection in AFP-negative patients (62).

There is a need for extensive studies on neoantigens as they can safely target tumors to achieve curative effects, unlike other existing methods. Additionally, it may also become a universal target antigen for immunotherapy in patients with different types of tumors. After adoptive cell therapy, a large number of tumor cells are destroyed in a short time in some patients, which will lead to fatal clinical events (63). In addition, T cells may kill all tumor or infected cells, such as in case of liver cancer caused by HBV infection, leading to serious organ function problems. This can be overcome by transferring suicide genes into T cells or transiently expressing TCR gene through mRNA electroporation (64).

### Expression of TCR

There are several challenges that remain, with respect to TCR expression, such as (i) to maximize the expression of TCR on the cell surface; (ii) to reduce the mismatch between the introduced TCR *α*- or *β*-chains and the endogenous TCR chains; (iii) to enhance the binding of TCR molecules to CD3 molecules; and (iv) to increase affinity between TCR and tumors (39). Studies have shown that the provision of additional CD3 molecules while giving T cell TCR genes can promote T cell affinity and anti-tumor activity *in vivo* (65). Besides, the liver is a special organ which has many immune suppression mechanisms (66), and may create obstacles in the future to immunotherapy for HCC.

### Immune Evasion and Immunosuppression

In addition, tumor immune evasion is also one of the factors that affect TCR-T cell therapy. The loss of autoantigen, loss of HLA molecules, and change of tumor antigen in the process rendered the T cells unable to recognize cancer cells (67, 68). The TCR-T cells derived from iPSCs are monoclonal. It was found that CD8 + T cells express LAG-3 and PD-1 molecules, leading to accelerated T cell depletion (69). Whether this phenomenon will appear in T cells derived from iPSCs, thus reducing its therapeutic effect remains to be seen. Issues with T cell homing is also one of the factors affecting adoptive immunotherapy. The nitration of CCL2 chemokines and the change of glycosylation of T cell surface glycoprotein are the factors that weaken T cell homing (70). Further, in some cases, despite the large number of specific CD8+ T cells, the tumor continues to grow (71). It may be due to inhibitory factors expressed by tumors, such as IL-10 and TGF-*β* (72) (**Table 2**).

**Table 2:** Current challenges, reasons and possible solutions of TCR-T induced by T-iPSCs for the treatment of Hepatocellular Carcinoma.

Challenges	Reasons	Possible solutions
Security	Off-target effects	Find neoantigens
	Targeted toxicity	
TCR mismatch	The introduced TCR chains match the endogenous TCR chains	A single viral vector encoding two TCR chains
		Gene knock-out
Tumor immune evasion	Loss of autoantigen	Find neoantigens
	Loss of HLA molecules	
	Change of tumor antigen	
	Monoclonal TCR-T	
T cell depletion	LAG-3 and PD-1 overexpression	Gene knock-out
		Targeted drugs
Problems with T cell homing	Nitration of CCL2 chemokines	Regulating the expression of chemokines
	T cell surface glycoprotein changed	
Liver autoimmune suppression	Regulatory myeloid populations maintain liver immune tolerance	Improve the immune microenvironment

TCR, T Cell Receptor; TCR-T, T cell receptor T-Cells; HLA, human leukocyte antigen; LAG-3, Lymphocyte activation gene-3; PD-1, Programmed Death-1; CCL2, chemokine ligand 2.

### Quality Control

Currently, advancement in the current protocols used to produce neoantigen T cells derived from T-iPSCs holds a great promise for regenerative medicine and therapeutic applications. Researchers have been able to produce functional neoantigen T cells from iPSCs and patient-derived tissues (73, 74). Sometimes, the quality of the patient’s T cell affects the production of T-iPSC, which is often not ideal. A study had developed a single-cell droplet microfluidic technology to screen functional TCR-T cells, which can overcome this problem (75). In 2020, HLA was used to transplant donor tissue or cells with the same HLA alleles into HLA haplotype heterozygous patients, which reduced the immune rejection reaction, thereby producing “off-the-shelf” T cells used to treat patients (76). The knock-out of TCRs and/or HLAs can help obtain universal TCR-T cells without MHC restriction. Researchers have been able to expand and scale the production of cells, and developed tools such as 3D thymic culture for mass production of neoantigen T cell. Moreover, development of 3D thymic organoids, coculture of multiple cytokines, and transplantation into 3D thymic organoids have improved the maturity and functionality of T cells. It is crucial to resolve challenges associated with genomic instability and formation of teratomas after transplantation of iPSC-derived T cells, in order to achieve its wide-scale clinical use in regenerative medicine and cell therapy.

## Conclusions and Future Perspectives

The immune system is an important line of defense for the body to resist external intrusions, supervisors and stabilize the internal environment. Antigen-antibody reactions are the essential means for the immune system to perform its functions. Tumor antigens play important roles in the occurrence and development of tumors and inducing the body’s anti-tumor immune responses. Therefore, whether in TCR-T cell therapy or other immunotherapies, finding, identifying and analyzing tumor antigens is the core of tumor immunology research. Among them, neoantigens are considered the most promising tumor antigens. The personalized immunotherapy model based on neoantigens generated by tumor-specific mutations is the main development direction of immunotherapy in solid tumors in the future.

In recent years, the rapid development of immunotherapy has become a new hope for mankind to fight tumors. Academics are of the opinion that the approach of using the immune system to attack tumors will become a turning point in cancer treatment. TCR-T cell therapy can be successfully applied to solid tumors, and has broad scope in the future. However, several challenges associated with the TCR-T cell therapy need to be resolved for its wider application. Improvement in the induction and differentiation of T-iPSCs, TCR-T cell production efficiency and quality, optimized TCR transfection system, transfection efficiency, TCR affinity, and cell expression levels can further improve T cell performance (72). In addition, progress in tumor immunotherapy methods depends on suitable immune targets, and the neoantigens generated by mutations are very ideal specific antigens. In view of the complex immunosuppressive microenvironment of tumors, neoantigen-based immunotherapy with a reasonable combination of immune checkpoint inhibitors and traditional tumor treatment modes such as radiotherapy, chemotherapy, and monoclonal antibodies targeting tumor antigens, can further increase the efficacy of immunotherapy and exert a more synergistic anti-tumor effect.

The induction and redifferentiation technology of iPSCs has gradually advanced, providing great convenience in the establishment of HCC treatment models and application in HCC immunotherapy. The use of T-iPSCs to produce a large number of highly individualized neoantigen specific TCR-T cells that can be used for treatment will also achieve practical application in the near future. Thymic organoids developed from patient-derived tissue have been shown to faithfully recapitulate the disease *in vitro* and could be a useful tool to study disease pathogenesis and screening of novel therapeutic drugs. However, currently, the high cost of preparation and complicated operating procedures of TCR-T cell therapy requires the need for further studies in the future to overcome these challenges.

In conclusion, researchers have been able to generate suitable neoantigen T cells derived from T-iPSCs to apply for clinical application. The optimal combination of TCR-T cell therapy and traditional HCC treatment methods also requires continuous exploration and research, striving to improve the efficacy while making it convenient for clinical application, so that more patients can benefit from it in the near future.

## Author Contributions

XL contributed significantly to analysis, fund support, and the conception of the review. FL and X-J-NM contributed to manuscript preparation and wrote the manuscript. W-LJ helped analyze and revised the manuscript. YL contributed to manuscript with some constructive suggestions. All authors contributed to the article and approved the submitted version.

## Funding

This work was supported in part by the Regional Project of National Natural Science Foundation of China (No. 82060119), Major Science and Technology Projects of Gansu Province (No. 1602FKDA001), and Gansu Science and Technology Program (No. 18JR2TA018).

## Conflict of Interest

The authors declare that the research was conducted in the absence of any commercial or financial relationships that could be construed as a potential conflict of interest.

## References

[1] Bray F Ferlay J Soerjomataram I Siegel RL Torre LA Jemal A. Global Cancer Statistics 2018: GLOBOCAN Estimates of Incidence and Mortality Worldwide for 36 Cancers in 185 Countries. CA Cancer J Clin (2018) 68(6):394–424. 10.3322/caac.21492 30207593

[2] Siegel RL Miller KD Jemal A. Cancer Statistics, 2019. CA Cancer J Clin (2019) 69(1):7–34. 10.3322/caac.21551 30620402

[3] Wang EA Stein JP Bellavia RJ Broadwell SR. Treatment Options for Unresectable HCC With a Focus on SIRT With Yttrium-90 Resin Microspheres. Int J Clin Pract (2017) 71(11):10. 10.1111/ijcp.12972 28758319

[4] Crocetti L Bargellini I Cioni R. Loco-Regional Treatment of HCC: Current Status. Clin Radiol (2017) 72(8):626–35. 10.1016/j.crad.2017.01.013 28258743

[5] Scarfo I Maus MV. Current Approaches to Increase CAR T Cell Potency in Solid Tumors: Targeting the Tumor Microenvironment. J Immunother Cancer (2017) 5:28. 10.1186/s40425-017-0230-9 28331617 PMC5359946

[6] Johnston MP Khakoo SI. Immunotherapy for Hepatocellular Carcinoma: Current and Future. World J Gastroenterol (2019) 25(24):2977–89. 10.3748/wjg.v25.i24.2977 PMC6603808 31293335

[7] Duffy AG Ulahannan SV Makorova-Rusher O Rahma O Wedemeyer H Pratt D. Tremelimumab in Combination With Ablation in Patients With Advanced Hepatocellular Carcinoma. J Hepatol (2017) 66(3):545–51. 10.1016/j.jhep.2016.10.029 PMC5316490 27816492

[8] Johnson LA June CH. Driving Gene-Engineered T Cell Immunotherapy of Cancer. Cell Res (2017) 27(1):38–58. 10.1038/cr.2016.154 28025979 PMC5223234

[9] Kalos M Levine BL Porter DL Katz S Grupp SA Bagg A. T Cells With Chimeric Antigen Receptors Have Potent Antitumor Effects and can Establish Memory in Patients With Advanced Leukemia. Sci Transl Med (2011) 3(95):95ra73. 10.1126/scitranslmed.3002842 PMC3393096 21832238

[10] Robbins PF Morgan RA Feldman SA Yang JC Sherry RM Dudley ME. Tumor Regression in Patients With Metastatic Synovial Cell Sarcoma and Melanoma Using Genetically Engineered Lymphocytes Reactive With NY-ESO-1. J Clin Oncol (2011) 29(7):917–24. 10.1200/JCO.2010.32.2537 PMC3068063 21282551

[11] Morgan RA Dudley ME Wunderlich JR Hughes MS Yang JC Sherry RM. Cancer Regression in Patients After Transfer of Genetically Engineered Lymphocytes. Science (New York NY) (2006) 314(5796):126–9. 10.1126/science.1129003 PMC2267026 16946036

[12] Johnson LA Morgan RA Dudley ME Cassard L Yang JC Hughes MS. Gene Therapy With Human and Mouse T-cell Receptors Mediates Cancer Regression and Targets Normal Tissues Expressing Cognate Antigen. Blood (2009) 114(3):535–46. 10.1182/blood-2009-03-211714 PMC2929689 19451549

[13] Singh N Kulikovskaya I Barrett DM Binder-Scholl G Jakobsen B Martinez D. T Cells Targeting NY-ESO-1 Demonstrate Efficacy Against Disseminated Neuroblastoma. Oncoimmunology (2016) 5(1):e1040216. 10.1080/2162402X.2015.1040216 26942053 PMC4760344

[14] Xia Y Tian X Wang J Qiao D Liu X Xiao L. Treatment of Metastatic non-Small Cell Lung Cancer With NY-ESO-1 Specific TCR Engineered-T Cells in a Phase I Clinical Trial: A Case Report. Oncol Lett (2018) 16(6):6998–7007. 10.3892/ol.2018.9534 30546433 PMC6256329

[15] Zhu W Peng Y Wang L Hong Y Jiang X Li Q. Identification of Alpha-Fetoprotein-Specific T-cell Receptors for Hepatocellular Carcinoma Immunotherapy. Hepatology (2018) 68(2):574–89. 10.1002/hep.29844 PMC7368991 29443377

[16] Rosenberg SA Restifo NP. Adoptive Cell Transfer as Personalized Immunotherapy for Human Cancer. Science (New York NY) (2015) 348(6230):62–8. 10.1126/science.aaa4967 PMC6295668 25838374

[17] Rapoport AP Stadtmauer EA Binder-Scholl GK Goloubeva O Vogl DT Lacey SF. NY-ESO-1-specific TCR-Engineered T Cells Mediate Sustained Antigen-Specific Antitumor Effects in Myeloma. Nat Med (2015) 21(8):914–21. 10.1002/hep.31662 PMC4529359 26193344

[18] Xie DY Ren ZG Zhou J Fan J Gao Q. Chinese Clinical Guidelines for the Management of Hepatocellular Carcinoma: Updates and Insights. Hepatobiliary Surg Nutr (2020) 9(4):452–63. 10.21037/hbsn-20-480 PMC7423548 32832496

[19] Chen L Qiao D Wang J Tian G Wang M. Cancer Immunotherapy With Lymphocytes Genetically Engineered With T Cell Receptors for Solid Cancers. Immunol Lett (2019) 216:51–62. 10.1016/j.imlet.2019.10.002 31597088

[20] Hafezi M Lin M Chia A Chua A Ho ZZ Fam R. Immunosuppressive Drug Resistant Armored TCR T Cells for Immune-Therapy of HCC in Liver Transplant Patients. Hepatology (2020) 2020:10.1002/hep.31662. 10.1002/hep.31662 33249625

[21] Schumacher TN Schreiber RD. Neoantigens in Cancer Immunotherapy. Science (New York NY) (2015) 348(6230):69–74. 10.1126/science.aaa4971 25838375

[22] Vogelstein B Papadopoulos N Velculescu VE Zhou S Diaz LA Kinzler KW. Cancer Genome Landscapes. Science (New York NY) (2013) 339(6127):1546–58. 10.1126/science.1235122 PMC3749880 23539594

[23] Robbins PF Lu Y-C El-Gamil M Li YF Gross C Gartner J. Mining Exomic Sequencing Data to Identify Mutated Antigens Recognized by Adoptively Transferred Tumor-Reactive T Cells. Nat Med (2013) 19(6):747–52. 10.1038/nm.3161 PMC3757932 23644516

[24] Tran E Turcotte S Gros A Robbins PF Lu Y-C Dudley ME. Cancer Immunotherapy Based on Mutation-Specific CD4+ T Cells in a Patient With Epithelial Cancer. Science (New York NY) (2014) 344(6184):641–5. 10.1126/science.1251102 PMC6686185 24812403

[25] Schumacher T Bunse L Pusch S Sahm F Wiestler B Quandt J. A Vaccine Targeting Mutant IDH1 Induces Antitumour Immunity. Nature (2014) 512(7514):324–7. 10.1038/nature13387 25043048

[26] Takahashi K Yamanaka S. Induction of Pluripotent Stem Cells From Mouse Embryonic and Adult Fibroblast Cultures by Defined Factors. Cell (2006) 126(4):663–76. 10.1016/j.cell.2006.07.024 16904174

[27] Takahashi K Tanabe K Ohnuki M Narita M Ichisaka T Tomoda K. Induction of Pluripotent Stem Cells From Adult Human Fibroblasts by Defined Factors. Cell (2007) 131(5):861–72. 10.1016/j.cell.2007.11.019 18035408

[28] Park IH Zhao R West JA Yabuuchi A Huo H Ince TA. Reprogramming of Human Somatic Cells to Pluripotency With Defined Factors. Nature (2008) 451(7175):141–6. 10.1038/nature06534 18157115

[29] Hamilton B Feng Q Ye M Welstead GG. Generation of Induced Pluripotent Stem Cells by Reprogramming Mouse Embryonic Fibroblasts With a Four Transcription Factor, Doxycycline Inducible Lentiviral Transduction System. J Vis Exp (2009) 2009(33):1447. 10.3791/1447 PMC3157852 19915522

[30] Karagiannis P Takahashi K Saito M Yoshida Y Okita K Watanabe A. Induced Pluripotent Stem Cells and Their Use in Human Models of Disease and Development. Physiol Rev (2019) 99(1):79–114. 10.1152/physrev.00039.2017 30328784

[31] Lai WH Ho JC Lee YK Ng KM Au KW Chan YC. ROCK Inhibition Facilitates the Generation of Human-Induced Pluripotent Stem Cells in a Defined, Feeder-, and Serum-Free System. Cell Reprogram (2010) 12(6):641–53. 10.1089/cell.2010.0051 PMC2993021 20858051

[32] Hubbard JJ Sullivan SK Mills JA Hayes BJ Torok-Storb BJ Ramakrishnan A. Efficient iPS Cell Generation From Blood Using Episomes and HDAC Inhibitors. J Vis Exp (2014) (92):e52009. 10.3791/52009 25408260 PMC4335579

[33] Okada M Oka M Yoneda Y. Effective Culture Conditions for the Induction of Pluripotent Stem Cells. Biochim Biophys Acta (2010) 1800(9):956–63. 10.1016/j.bbagen.2010.04.004 20417254

[34] Nakagawa M Koyanagi M Tanabe K Takahashi K Ichisaka T Aoi T. Generation of Induced Pluripotent Stem Cells Without Myc From Mouse and Human Fibroblasts. Nat Biotechnol (2008) 26(1):101–6. 10.1038/nbt1374 18059259

[35] Wernig M Meissner A Cassady JP Jaenisch R. c-Myc is Dispensable for Direct Reprogramming of Mouse Fibroblasts. Cell Stem Cell (2008) 2(1):10–2. 10.1016/j.stem.2007.12.001 18371415

[36] Nishimura T Kaneko S Kawana-Tachikawa A Tajima Y Goto H Zhu D. Generation of Rejuvenated Antigen-Specific T Cells by Reprogramming to Pluripotency and Redifferentiation. Cell Stem Cell (2013) 12(1):114–26. 10.1016/j.stem.2012.11.002 23290140

[37] Gattinoni L Klebanoff CA Restifo NP. Paths to Stemness: Building the Ultimate Antitumour T Cell. Nat Rev Cancer (2012) 12(10):671–84. 10.1038/nrc3322 PMC6352980 22996603

[38] Minagawa A Yoshikawa T Yasukawa M Hotta A Kunitomo M Iriguchi S. Enhancing T Cell Receptor Stability in Rejuvenated iPSC-Derived T Cells Improves Their Use in Cancer Immunotherapy. Cell Stem Cell (2018) 23(6):850–8 e4. 10.1016/j.stem.2018.10.005 30449714

[39] Thomas S Stauss HJ Morris EC. Molecular Immunology Lessons From Therapeutic T-cell Receptor Gene Transfer. Immunology (2010) 129(2):170–7. 10.1111/j.1365-2567.2009.03227.x PMC2814459 20561357

[40] Okamoto S Mineno J Ikeda H Fujiwara H Yasukawa M Shiku H. Improved Expression and Reactivity of Transduced Tumor-Specific TCRs in Human Lymphocytes by Specific Silencing of Endogenous TCR. Cancer Res (2009) 69(23):9003–11. 10.1158/0008-5472.CAN-09-1450 19903853

[41] Provasi E Genovese P Lombardo A Magnani Z Liu PQ Reik A. Editing T Cell Specificity Towards Leukemia by Zinc Finger Nucleases and Lentiviral Gene Transfer. Nat Med (2012) 18(5):807–15. 10.1038/nm.2700 PMC5019824 22466705

[42] Crompton JG Clever D Vizcardo R Rao M Restifo NP. Reprogramming Antitumor Immunity. Trends Immunol (2014) 35(4):178–85. 10.1016/j.it.2014.02.003 PMC4373650 24661777

[43] Vizcardo R Masuda K Yamada D Ikawa T Shimizu K Fujii S. Regeneration of Human Tumor Antigen-Specific T Cells From iPSCs Derived From Mature CD8(+) T Cells. Cell Stem Cell (2013) 12(1):31–6. 10.1016/j.stem.2012.12.006 23290135

[44] Kawamoto H Masuda K Nagano S Maeda T. Cloning and Expansion of Antigen-Specific T Cells Using iPS Cell Technology: Development of “Off-the-Shelf” T Cells for the Use in Allogeneic Transfusion Settings. Int J Hematol (2018) 107(3):271–7. 10.1007/s12185-018-2399-1 29388165

[45] Bareli R Cohen CJ. MHC-Multimer Guided Isolation of Neoepitopes Specific T Cells as a Potent-Personalized Cancer Treatment Strategy. Oncoimmunology (2016) 5(7):e1159370. 10.1080/2162402X.2016.1159370 27622017 PMC5006892

[46] Cohen CJ Gartner JJ Horovitz-Fried M Shamalov K Trebska-McGowan K Bliskovsky VV. Isolation of Neoantigen-Specific T Cells From Tumor and Peripheral Lymphocytes. J Clin Invest (2015) 125(10):3981–91. 10.1172/JCI82416 PMC4607110 26389673

[47] Montel-Hagen A Crooks GM. From Pluripotent Stem Cells to T Cells. Exp Hematol (2019) 71:24–31. 10.1016/j.exphem.2018.12.001 30590093

[48] Vizcardo R Klemen ND Islam SMR Gurusamy D Tamaoki N Yamada D. Generation of Tumor Antigen-Specific iPSC-Derived Thymic Emigrants Using a 3D Thymic Culture System. Cell Rep (2018) 22(12):3175–90. 10.1016/j.celrep.2018.02.087 PMC5930030 29562175

[49] Chen X Lei F Wang L Xiong X Song J. Generation of Tumor Antigen-Specific Cytotoxic T Lymphocytes From Pluripotent Stem Cells. Methods Mol Biol (2019) 1884:43–55. 10.1007/978-1-4939-8885-3_3 30465194 PMC6629431

[50] Chen YT Boyer AD Viars CS Tsang S Old LJ Arden KC. Genomic Cloning and Localization of CTAG, a Gene Encoding an Autoimmunogenic Cancer-Testis Antigen NY-ESO-1, to Human Chromosome Xq28. Cytogenet Cell Genet (1997) 79(3-4):237–40. 10.1159/000134734 9605863

[51] Fujita S Wada H Jungbluth AA Sato S Nakata T Noguchi Y. NY-ESO-1 Expression and Immunogenicity in Esophageal Cancer. Clin Cancer Res (2004) 10(19):6551–8. 10.1158/1078-0432.CCR-04-0819 15475443

[52] Jungbluth AA Antonescu CR Busam KJ Iversen K Kolb D Coplan K. Monophasic and Biphasic Synovial Sarcomas Abundantly Express Cancer/Testis Antigen NY-ESO-1 But Not MAGE-A1 or CT7. Int J Cancer (2001) 94(2):252–6. 10.1002/ijc.1451 11668506

[53] Ries J Mollaoglu N Vairaktaris E Neukam FW Nkenke E. Diagnostic and Therapeutic Relevance of NY-ESO-1 Expression in Oral Squamous Cell Carcinoma. Anticancer Res (2009) 29(12):5125–30. 10.1155/2008/359840 20044626

[54] Jäger E Chen YT Drijfhout JW Karbach J Ringhoffer M Jäger D. Simultaneous Humoral and Cellular Immune Response Against Cancer-Testis Antigen NY-ESO-1: Definition of Human Histocompatibility Leukocyte Antigen (HLA)-A2-binding Peptide Epitopes. J Exp Med (1998) 187(2):265–70. 10.1084/jem.187.2.265 PMC2212106 9432985

[55] Jäger E Gnjatic S Nagata Y Stockert E Jäger D Karbach J. Induction of Primary NY-ESO-1 Immunity: CD8+ T Lymphocyte and Antibody Responses in Peptide-Vaccinated Patients With NY-ESO-1+ Cancers. Proc Natl Acad Sci U S A (2000) 97(22):12198–203. 10.1073/pnas.220413497 PMC17318 11027314

[56] Parkhurst MR Yang JC Langan RC Dudley ME Nathan D-AN Feldman SA. T Cells Targeting Carcinoembryonic Antigen can Mediate Regression of Metastatic Colorectal Cancer But Induce Severe Transient Colitis. Mol Ther J Am Soc Gene Ther (2011) 19(3):620–6. 10.1038/mt.2010.272 PMC3048186 21157437

[57] Palmer DC Chan C-C Gattinoni L Wrzesinski C Paulos CM Hinrichs CS. Effective Tumor Treatment Targeting a Melanoma/Melanocyte-Associated Antigen Triggers Severe Ocular Autoimmunity. Proc Natl Acad Sci U S A (2008) 105(23):8061–6. 10.1073/pnas.0710929105 PMC2409137 18523011

[58] Morgan RA Chinnasamy N Abate-Daga D Gros A Robbins PF Zheng Z. Cancer Regression and Neurological Toxicity Following Anti-MAGE-A3 TCR Gene Therapy. J Immunother (2013) 36(2):133–51. 10.1097/CJI.0b013e3182829903 PMC3581823 23377668

[59] Tan EM Zhang J. Autoantibodies to Tumor-Associated Antigens: Reporters From the Immune System. Immunol Rev (2008) 222:328–40. 10.1111/j.1600-065X.2008.00611.x PMC2719766 18364012

[60] Zhang JY Tan EM. Autoantibodies to Tumor-Associated Antigens as Diagnostic Biomarkers in Hepatocellular Carcinoma and Other Solid Tumors. Expert Rev Mol Diagn (2010) 10(3):321–8. 10.1586/erm.10.12 PMC2896686 20370589

[61] Koziol JA Imai H Dai L Zhang JY Tan EM. Early Detection of Hepatocellular Carcinoma Using Autoantibody Profiles From a Panel of Tumor-Associated Antigens. Cancer Immunol Immunother (2018) 67(5):835–41. 10.1007/s00262-018-2135-y PMC5930126 29497780

[62] Wang K Li M Qin J Sun G Dai L Wang P. Serological Biomarkers for Early Detection of Hepatocellular Carcinoma: A Focus on Autoantibodies Against Tumor-Associated Antigens Encoded by Cancer Driver Genes. Cancers (Basel) (2020) 12(5):1271. 10.3390/cancers12051271 PMC7280966 32443439

[63] MacKay M Afshinnekoo E Rub J Hassan C Khunte M Baskaran N. The Therapeutic Landscape for Cells Engineered With Chimeric Antigen Receptors. Nat Biotechnol (2020) 38(2):233–44. 10.1038/s41587-019-0329-2 31907405

[64] Bertoletti A Tan AT. Challenges of CAR- and TCR-T Cell-Based Therapy for Chronic Infections. J Exp Med (2020) 217(5):e20191663. 10.1084/jem.20191663 32163104 PMC7201928

[65] Ahmadi M King JW Xue S-A Voisine C Holler A Wright GP. CD3 Limits the Efficacy of TCR Gene Therapy In Vivo. Blood (2011) 118(13):3528–37. 10.1182/blood-2011-04-346338 21750319

[66] Robinson MW Harmon C O’Farrelly C. Liver Immunology and its Role in Inflammation and Homeostasis. Cell Mol Immunol (2016) 13(3):267–76. 10.1038/cmi.2016.3 PMC4856809 27063467

[67] Arcangeli S Mestermann K Weber J Bonini C Casucci M Hudecek M. Overcoming Key Challenges in Cancer Immunotherapy With Engineered T Cells. Curr Opin Oncol (2020) 32(5):398–407. 10.1097/CCO.0000000000000664 32796230

[68] Liu M Guo F. Recent Updates on Cancer Immunotherapy. Precis Clin Med (2018) 1(2):65–74. 10.1093/pcmedi/pby011 30687562 PMC6333045

[69] Matsuzaki J Gnjatic S Mhawech-Fauceglia P Beck A Miller A Tsuji T. Tumor-Infiltrating NY-ESO-1-specific CD8+ T Cells are Negatively Regulated by LAG-3 and PD-1 in Human Ovarian Cancer. Proc Natl Acad Sci U S A (2010) 107(17):7875–80. 10.1073/pnas.1003345107 PMC2867907 20385810

[70] Manfredi F Cianciotti BC Potenza A Tassi E Noviello M Biondi A. TCR Redirected T Cells for Cancer Treatment: Achievements, Hurdles, and Goals. Front Immunol (2020) 11:1689. 10.3389/fimmu.2020.01689 33013822 PMC7494743

[71] Rosenberg SA Sherry RM Morton KE Scharfman WJ Yang JC Topalian SL. Tumor Progression can Occur Despite the Induction of Very High Levels of Self/Tumor Antigen-Specific CD8+ T Cells in Patients With Melanoma. J Immunol (2005) 175(9):6169–76. 10.4049/jimmunol.175.9.6169 16237114

[72] Zhang L Morgan RA. Genetic Engineering With T Cell Receptors. Adv Drug Deliv Rev (2012) 64(8):756–62. 10.1016/j.addr.2011.11.009 PMC3404458 22178904

[73] Maeda T Nagano S Kashima S Terada K Agata Y Ichise H. Regeneration of Tumor-Antigen-Specific Cytotoxic T Lymphocytes From iPSCs Transduced With Exogenous TCR Genes. Mol Ther Methods Clin Dev (2020) 19:250–60. 10.1016/j.omtm.2020.09.011 PMC7566080 33102617

[74] Maeda T Nagano S Ichise H Kataoka K Yamada D Ogawa S. Regeneration of CD8αβ T Cells From T-cell-Derived iPSC Imparts Potent Tumor Antigen-Specific Cytotoxicity. Cancer Res (2016) 76(23):6839–50. 10.1158/0008-5472.CAN-16-1149 27872100

[75] Segaliny AI Li G Kong L Ren C Chen X Wang JK. Functional TCR T Cell Screening Using Single-Cell Droplet Microfluidics. Lab Chip (2018) 18(24):3733–49. 10.1039/C8LC00818C PMC6279597 30397689

[76] Nagano S Maeda T Ichise H Kashima S Ohtaka M Nakanishi M. High Frequency Production of T Cell-Derived iPSC Clones Capable of Generating Potent Cytotoxic T Cells. Mol Ther Methods Clin Dev (2020) 16:126–35. 10.1016/j.omtm.2019.12.006 PMC6965501 31970197

